# The application of autologous cancer immunotherapies in the age of memory-NK cells

**DOI:** 10.3389/fimmu.2023.1167666

**Published:** 2023-05-02

**Authors:** Gaby D. Lizana-Vasquez, Madeline Torres-Lugo, R. Brent Dixon, John D. Powderly, Renaud F. Warin

**Affiliations:** ^1^ Department of Chemical Engineering, University of Puerto Rico-Mayagüez, Mayagüez, Puerto Rico; ^2^ Cancer Research Clinic, Carolina BioOncology Institute (CBOI), Huntersville, NC, United States; ^3^ Human Applications Lab (HAL) - BioCytics, Huntersville, NC, United States

**Keywords:** autologous immunotherapy, solid tumor, memory-like natural killer cells, cellular stress, upscale production, point-of-care manufacturing

## Abstract

Cellular immunotherapy has revolutionized the oncology field, yielding improved results against hematological and solid malignancies. NK cells have become an attractive alternative due to their capacity to activate upon recognition of “stress” or “danger” signals independently of Major Histocompatibility Complex (MHC) engagement, thus making tumor cells a perfect target for NK cell-mediated cancer immunotherapy even as an allogeneic solution. While this allogeneic use is currently favored, the existence of a characterized memory function for NK cells (“memory-like” NK cells) advocates for an autologous approach, that would benefit from the allogeneic setting discoveries, but with added persistence and specificity. Still, both approaches struggle to exert a sustained and high anticancer effect *in-vivo* due to the immunosuppressive tumor micro-environment and the logistical challenges of cGMP production or clinical deployment. Novel approaches focused on the quality enhancement and the consistent large-scale production of highly activated therapeutic memory-like NK cells have yielded encouraging but still unconclusive results. This review provides an overview of NK biology as it relates to cancer immunotherapy and the challenge presented by solid tumors for therapeutic NKs. After contrasting the autologous and allogeneic NK approaches for solid cancer immunotherapy, this work will present the current scientific focus for the production of highly persistent and cytotoxic memory-like NK cells as well as the current issues with production methods as they apply to stress-sensitive immune cells. In conclusion, autologous NK cells for cancer immunotherapy appears to be a prime alternative for front line therapeutics but to be successful, it will be critical to establish comprehensives infrastructures allowing the production of extremely potent NK cells while constraining costs of production.

## 1 Introduction

Immunotherapy is a therapeutic approach that harnesses the immune system against cancer. It has revolutionized the oncology field in terms of effectiveness and offers personalized, targeted treatment options that ultimately yield improved results when compared to surgery, chemotherapy, and/or radiation treatments. The key attributes of cancer immunotherapy are detecting, surveilling, and destroying neoplasm cells using immune cells. To date, six cellular therapy products, all autologous CAR-T cells against hematological malignancies, have been approved by the U.S. Food and Drug Administration (FDA) (https://www.cancer.gov). These treatments require a tailored product for each individual patient. As a consequence of the successful results obtained with CAR-T cells, the use and engineering potentiation to fight cancer of different immune cell types (1), such as dendritic cells, macrophages, and natural killer (NK) cells, has seen increased interest (2).

NK cells, monocytes, macrophages, and to some degree, dendritic cells constitute the innate lymphoid cell family, which is the first line of defense against invasive pathogens and transformed cells in the human body (3, 4). The discovery and characterization of NK cells dates back to 1975 when Herberman et al. and Kiessling et al. found a natural cytotoxic activity of a subpopulation of lymphoid cells against cancer cells in mouse studies (5, 6). The use of innate lymphocytes NK cells is attractive because of their unique ability to recognize cancer cells and exert antitumor cell cytotoxicity (7). Innate NK cells offer a very attractive approach to cancer therapy. Since NK cells base their initial recognition on particular “stress” or “danger” signals, tumor cells become a perfect target for NK cells.

**Figure 1:**
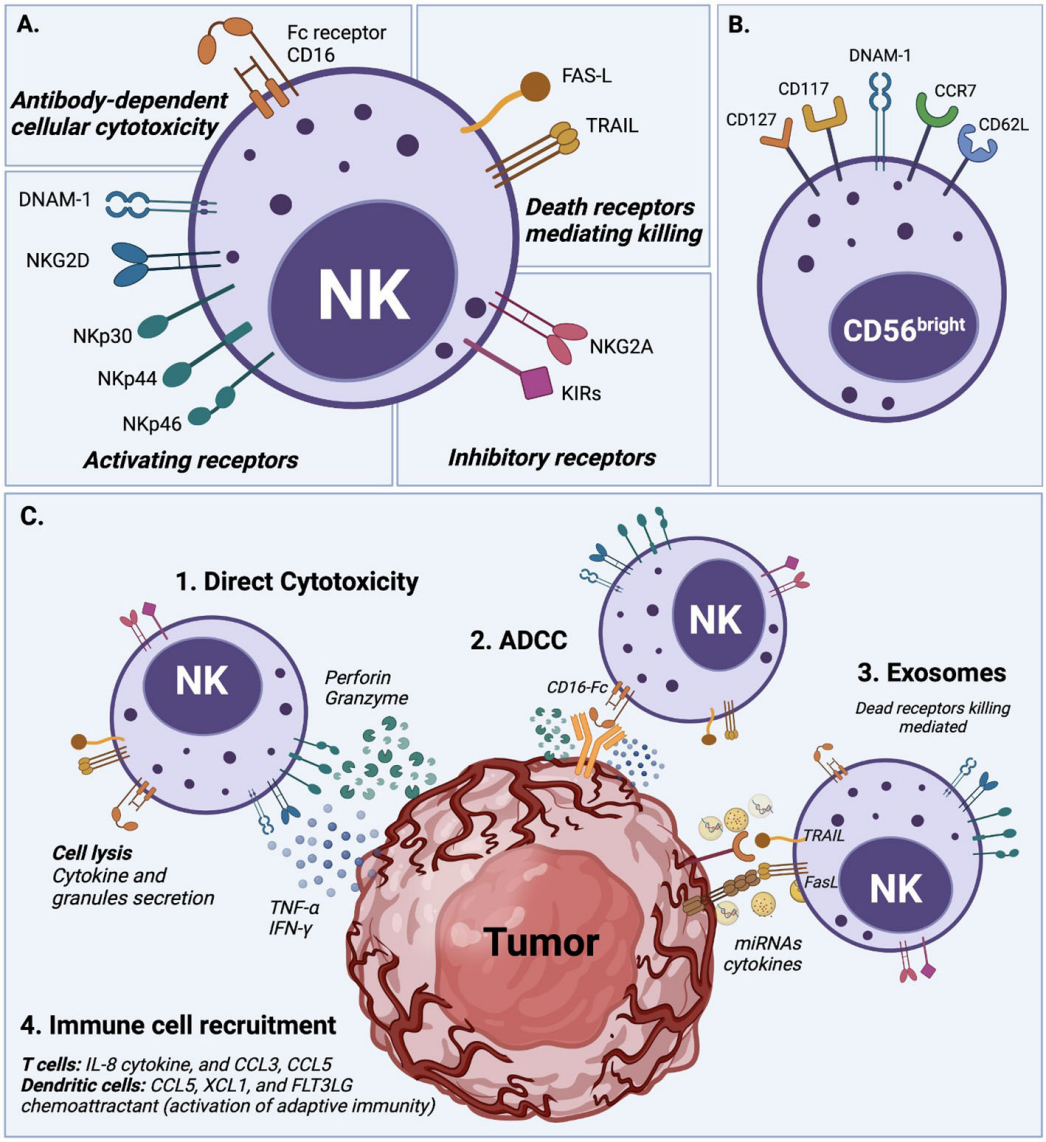
*NK cell activation and natural cytotoxicity.* **(A)** NK cells have a natural ability to recognize abnormal cells and exert cell cytotoxicity through different mechanisms. (1) NK cells exert direct cytotoxicity against compromised cells mainly by the secretion of IFN-γ and TNF-α cytokines, and the release of perforin and granzyme cytolytic granules. (2) Antibody-dependent cellular cytotoxicity (ADCC) is another mechanism of NK cell cytotoxicity, which is mediated by the CD16 (FcRIII) receptor after recognizing and bound target cells. (3) NK cells display dead receptors, FAS-L and TRAIL, and exert their killing capability against compromised cells through the release of exosome that contains small non-coding RNAs (miRNAs) and cytokines (8–10). (4) The immune cell recruitment of T cells and dendritic cells also plays an essential role in NK cell cytotoxicity to enhance their action through soluble cytokine IL-2 and chemokine (CCL3, CCL5, XCL1, and FLT3LG) secretion. **(B)** Overview of NK cell receptors. All described cytotoxicity mechanisms are in response to an orchestrated balance of activating receptors (e.g., DNAM-1, NKG2D, NKp30, NKp44, NKp46) and inhibitory receptors (e.g., NKG2A, KIRs family) that determine the NK cell activation state. **(C)** Phenotype of circulating CD56^bright^ NK cells which typically express DNAM-1, CD62L (L-selectin), and CCR7, but also in some cases CD117 (c-kit), and CD127 (IL-7Rα).

NK cell cytotoxicity is exerted through a delicate balance mechanism of activating and inhibiting surface receptors, without an antibody or MHCI-strict dependence, as detailed in **Figure 1** (1, 8, 11). The lack of expression of CD3 characterizes the human NK cell population, but it expresses the CD56 and CD16 cell surface markers and is classified into two main subsets (3). CD56^bright^CD16^lo/-^ NK cells are poorly cytotoxic and mainly produce pro-inflammatory cytokines after cytokine stimulation. CD56^dim^CD16^+^ NK cells, which represent about 90% of peripheral blood NK cells, are highly cytotoxic, but expand poorly and do not show a significant *de-novo* cytokine production (3, 12). Unsurprisingly CD16, also known as FcγRIIIA, was characterized early as critical to the proper activation of the ADCC (Antibody-Dependent Cell Cytotoxicity) response (13). These NK cell subsets also possess metabolic differences; for example, under cytokine stimulation, the CD56^bright^ subset is more susceptible to upregulating expression of nutrients receptors, amino acid transporters, and transferring receptors than the CD56^dim^ subset (12, 14, 15). However, several studies agree that both subsets, CD56^bright^ and CD56^dim^, can be cytotoxic or produce cytokines after suitable *in vitro* stimulation (3).

Target cell recognition and NK cell activation occur through different mechanisms (see **Figure 1**). For example, target cells can be recognized through the overexpression of ligands for the NK cell activating receptors such as natural killer group 2D (NKG2D), costimulatory receptors (DNAX accessory molecule-1 DNAM-1), and natural cytotoxicity receptors (NKp46). The ligands for these receptors are MHC class I chain-related MIC or UL16-Binding Proteins ULBPs (16), Nectin-2 or Poliovirus Receptor PVR (17), and B7H6 (18), respectively. Another target cell recognition mechanism is through a low-level expression of ligands for NK cell inhibitory receptors (e.g., KIRs family) such as MHC-I or the non-classical HLA-E for NKG2A (8, 19, 20). NK cells will kill cells that express low levels of MHC-I or HLA-E. Therefore, cancer cells that downregulate MHC-I molecules to evade T cell cytotoxicity can be targets for NK cells (21). Once target cells are recognized, NK cells exert their cytotoxicity by secreting cytokines and chemokines. They release perforin and granzyme cytolytic granules upon direct contact with the target malignant cell to cause cell apoptosis. NK cells also generate interferon-gamma (IFN-γ) and tumor necrosis factor-alpha (TNF-α), which exert antitumor effects and upregulate other immune responses (11, 19, 22).

All these features make NK cells particularly useful for autologous and allogeneic cancer immunotherapy, since they were recognized early on for their potential use in the allogeneic setting as an “off-the-shelf” product (23). Still, some challenges are standing before a successful anticancer treatment can be implemented, including short-term persistence, sensitivity, and clinical-grade *ex vivo* expansion (24). Despite the encouraging results of NK cells against hematological malignancies and solid tumors, the tumor microenvironment presents a steep challenge to the success of cancer immunotherapies. In the past decade, it was found that NK cells can exhibit memory-like functions under certain circumstances. This memory function can provide superior host protection compared to naive NK cells, due to their faster recognition and higher cytotoxic responses against tumor cells (25, 26). It could be a powerful property if successfully leveraged against solid tumors.

In this review, we will provide the traditional overview of the use of NK cells in cancer immunotherapy along with their autologous and allogeneic approaches. Allogeneic NK cell therapy dominates the adoptive NK cell-mediated cancer immunotherapy. Despite this fact, ongoing research and some clinical trials with autologous settings suggest its use as an alternative when the allogeneic setting is not applicable or unavailable (27). Then, the characteristics of NK cells memory function and the memory-like NK cell production for NK cell-mediated cancer immunotherapy will be described. Finally, the enhancement of non-genetically modified autologous NK cells will be shown along with their cancer therapeutic advantages when compared to genetically engineered or allogeneic cells.

## 2 NK cells in cancer immunotherapy

Since the identification of the NK cell population in the 1970s (5, 6), this immune cell lineage has been used as an anticancer treatment because of its capability to identify and kill malignant cells without priming, in an MHC-I non-restricted manner (28–30). In contrast to T cells, NK cells do not need prior antigen priming by MHC-I molecules to mediate their killing capability (28, 30), instead relying on a state of equilibrium that can be influenced by recognition of multiple markers of “missing self” (missing classical of non-classical MHC markers) or “induced self-recognition” (induced markers of abnormal-self, for example generated upon virus infection). The use of NK cells to fight hematological malignancies, including leukemia, lymphoma, and multiple myeloma, yielded encouraging results in pre-clinical and clinical studies (31). These favorable outcomes stem from different mechanisms that NK cells exert against tumor cells. In normal conditions, healthy cells of the human body have either absent or low expression of ligands for activating receptors. When compromised, stressed, or modified, cells upregulate these ligands, becoming more sensitive to NK cells (32). This mechanism, along with the tolerance of a KIR-HLA mismatch between recipient and donors, makes NK cells suitable for an “off-the-shelf” allogeneic cancer immunotherapy product. This allogeneic approach is based on the NK cells’ ability to recognize, direct, and rapidly lyse cancerous, stressed, and viral-infected cells without MHC-dependent priming (33–35). It avoids the rejection of self-MHC recognition and has a low risk of cytokine release syndrome (“cytokine storm”), graft-versus-host disease (GvHD), and other immune-associated off-target events observed with CAR-T cells (2, 24). Additionally, it was corroborated that autologous and allogeneic NK cells are a fantastic alternative therapy when cancer cells become resistant to chemotherapy, T cells, or checkpoint immunotherapy (29, 30, 36).

Allogeneic NK cell immunotherapy has been investigated in several clinical trials. It has been used not only against hematologic malignancies, but also against solid tumors, yielding encouraging results even with the immunocompromising tumor microenvironment. Melanoma, neuroblastoma, breast cancer, hepatocellular cancer, ovarian cancer, renal cell carcinoma, and colorectal cancer are examples of treated solid tumors (1, 4, 37). To date, more than 150 clinical trials of NK cells against cancer have been developed. **Table 1** illustrates 15 out of 150 trials of NK cells against cancer. These 15 trials explicitly focused on fighting solid tumors using allogeneic cells; most of them were completed, but two were terminated due to toxicity (NCT00582816) or the death of 2 patients (NCT01337544).

**Table 1:** Allogeneic NK cells clinical trials against solid tumors.

Identifier	Condition or disease	Additional treatment	Study phase	Recruitment Status	Participants	Initial date	Goals and Outcome
NCT00640796	Ewing sarcoma family of tumors (ESFT) and rhabdomyosarcoma (RMS)	Chemotherapy	I	Completed	22	September 2008	Determine the maximum tolerable dose of expanded NK cells in patients. No results have been posted.
NCT00582816	Solid Tumors	Chemotherapy	I/II	Terminated (toxicity)	9	August 2008	Engraftment Failure was reported in 3 patients within 28 of therapy. Many patients developed grade III or IV GVHD.
NCT00823524	Brain and Central Nervous System Tumors	HLA-haploidentical hematopoietic cell transplantation	I/II	Completed	47	January 2009	Evaluation of safety and side effects of donor NK cell infusion. No results have been posted.
NCT00855452	Metastatic Breast Cancer Malignant Melanoma Renal Cell Cancer Gastrointestinal Cancer	NA	II	Completed	20	January 2009	Evaluation of cancer progression on days 7, 17, and 28 post-cell therapy. No results have been posted.
NCT01212341	Solid Tumors	NA	I	Completed	18	September 2010	Determine the MTD of allogeneic NK cells within 4 to 5 weeks. No results have been posted.
NCT01287104	Solid Tumors	Stem Cell Infusion		Completed	34	January 2011	Five of 9 transplant recipients experienced acute GVHD following therapy (grade 4 was observed in 3 patients) (38).
NCT01337544	Childhood Solid Tumor	Haploidentical Stem Cell Transplantation	I	Terminated	6	January 2011	Measure safety and tolerability of the therapy. No results have been posted and the study was halted due to a claim against the hospital by parents of two patients who died.
NCT01875601	Solid Tumors Brain Tumors Sarcoma Neuroblastoma	RhIL-15	I	Completed	16	June 2013	Assess the toxicity of infusing escalating doses of autologous NK cells and the feasibility of harvesting and expanding activated NK cells to meet the proposed escalating dose. No results have been posted.
NCT02100891	Ewing Sarcoma Neuroblastoma Rhabdomyosarcoma Osteosarcoma CNS Tumors	NA	II	Active, not recruiting		March 2014	Disease control rate and overall survival after 6 months and 1 year cell therapy respectively. No results have been posted.
NCT02130869	Neuroblastoma High-risk Tumor	CD133+ autologous stem cell infusion Several drugs	I	Completed	8	October 2014	Determine the positive ANC engraftment in patients. No results have been posted.
NCT03213964	Epithelial Ovarian Cancer Fallopian Tube Cancer Primary Peritoneal Cancer	IL-2	I	Completed	10	October 2017	Determine the maximum tolerated dosage of NK cell therapy in patients. No results have been posted.
NCT03319459	Gastric Cancer Colorectal Cancer Melanoma Pancreatic Cancer Breast Cancer	Cetuximab Trastuzumab	I	Completed	44	January 2018	Determine the incidence of DLT within each dose, 28 days after administration. No results have been posted.
NCT02857920	Solid Tumors	Bevacizumab	I/II	Completed	45	August 2016	Determine the PFS and OS in patients over 1 and 3 years respectively. No results have been posted.
NCT02853903	Malignant Solid Tumor	NA	II	Completed	20	August 2016	Determine the relief degree of tumors within 3 months. No results have been posted.
NCT03420963	Relapsed or Refractory solid tumors	Cyclophosphamide Etoposide	I	Recruiting	38	August 2021	Determine the incidence of adverse events and maximum tolerated dose of expanded allogeneic NK cells. No results have been posted.

PFS, progress-free survival; OS, overall survival; DLT, dose-limiting toxicities; ANC, absolute neutrophil count; MTD, maximum tolerable dose. Source: https://clinicaltrials.gov.

## 3 Autologous NK therapy against cancer

The principle of allogeneic transplant has been explored since the early 1950s (39), and allogeneic therapies are defined by the transplant of biological material from a donor to a host in order to cure a disease. This approach has been intensively sought by researcher as a tool against cancer since it would allow for an “off-the-shelve”, universal solution (40). NK cells are a privileged tool for allogeneic immunotherapies mainly because, unlike cytotoxic T-Cells, their MHC independence does not trigger Graft *vs*. Host Disease (GvHD) (41). Because of this obvious advantage, the consideration to use the own patient NK cells (autologous setting) has received little attention. Ongoing research with autologous settings suggests it is a promising strategy when allogeneic cells are limited or unavailable (27). Autologous products are simpler to obtain and avoid some challenges of human leukocyte antigen (HLA) mismatched cellular therapy, such as the failure to persist for more than a few weeks in an immune incompatible environment (42–44). Another example is the off-target activation of allogeneic cell products due to the abundance of targets in the non-immune compatible recipient that can result in NK cell misdirection, exhaustion, or anergy (44). Additionally, the use of chemotherapy is needed before allogeneic therapy injection to avoid immunological rejection of the recipient (42, 45). **Table 2** summarizes the advantages and limitations offered by both types of NK cell therapy, autologous and allogeneic settings.

**Table 2:** NK cell therapies comparison.

Autologous	Allogeneic
HLA compatibility	KIR-HLA mismatch with donor may trigger alloreactivity of NK cells
Low risk of getting graft versus host disease	Potential risk of getting graft versus host disease
Faces a known environment	Non-immune incompatible environment
Designed to be used in the same patient	Can be used with any recipient (“off-the-shelf” product)
Compatible environment facilitates the NK cell expansion and persistence	More probability of reaching cell exhaustion or anergy
No need to use immunosuppressive regimen	To avoid immunological rejection, chemotherapy is needed before the therapy
The cell source is the patient, who usually possesses cells with reduced effector function (“exhausted”)	Wide range of NK cells sources

The initial clinical autologous NK cell therapy was conducted in 2003 and used IL-2 *ex vivo* cytokine-activated NK cells. The results of this trial did not produce significant improvements in patient disease outcomes of lymphoma and breast cancer (46). More recently, Nahi et al. performed multiple infusion doses of *ex vivo* activated and expanded autologous NK cells in multiple myeloma (MM) patients. This treatment resulted in a reduction or minimal residual disease with increased NK cell circulation and granzyme B levels, persisting for several weeks after the last infusion (27). Another clinical trial used autologous NK cells combined with Bortezomib against different solid tumors such as Chronic Myeloid Leukemia (CML), MM, carcinoma, pancreatic, colon/rectal, and non-small-cell lung cancer (NCT00720785). Also, 12 young patients with recurrent or refractory brain tumors were the target in a phase I clinical trial (NCT02271711) that evaluated autologous *ex vivo* expanded NK cells. These two trials yielded no conclusive data.

In another study, patients with metastatic digestive cancer, whose standard therapy failed, were enrolled in a phase I clinical trial to be treated with *ex vivo* expanded NK cells (UMIN000007527). Expanded and γ-irradiated PBMCs were used as feeder cells to stimulate NK-cell expansion. Injected NK cells were demonstrated to have high *in vitro* lytic action and strong expression of NKG2D and CD16. Even when no clinical responses were observed, the therapy did not show severe adverse events, and the cytotoxicity of peripheral NK cells was elevated up to 4 weeks following the last transfusion (47). Bae et al. performed a phase I clinical trial of autologous expanded and activated NK cell therapy. Cells were administered for 5 consecutive days in a dose-escalating manner combined with chemotherapy in 11 hepatocellular carcinoma patients. They observed a disease control rate of 81.8% with no decompensation or adverse events (48). Similarly, pediatric medulloblastoma and ependymoma were treated with intraventricular infusions of *ex vivo* expanded autologous NK cells. NK cells were expanded from PBMCs by co-culturing with irradiated K562 feeder cells genetically modified to express costimulatory molecules. In this case, 8 of the 9 patients still showed progressing disease. Nevertheless, NK cells increased in the cerebrospinal fluid when higher dose levels with repetitive infusions were used. The authors suggested that these findings support additional studies of NK cell infusions against brain malignancies in children (49).

Although many strategies efficiently generate large quantities of expanded NK cells *ex vivo*, it is still unclear whether the expanded NK cells can persist/proliferate *in vivo* without exogenous human cytokines. Vahedi et al., in a pre-clinical study with humanized mice, demonstrated that autologous human immune cells support the *in vivo* survival of *ex vivo* expanded human NK cells. They administrated *ex vivo* expanded human cord blood (HCB) derived NK cells into humanized mice reconstituted with autologous HCB immune cells. In contrast to the control mice lacking human immune cells, NK cells could survive and possibly proliferate *in vivo* without exogenous cytokine administration (50). This study underlines the benefits of the autologous setting to maintain the correct immune homeostasis and feedback mechanisms.

Despite these efforts, the success of autologous NK cell therapies has been affected by the aggressiveness of malignant cells, tumor escape, and manufacturing letdowns due to the low number and compromised function of patient-derived NK cells. For that reason, several approaches emerged to enhance their antibody-dependent cellular cytotoxicity (ADCC). Some examples include the addition of checkpoint receptor blockers, antitumor monoclonal antibodies, bi-/tri-specific killer engagers (BiKEs and TriKEs), and the use of memory-type NK cells, which showed beneficial effects (51). Additionally, therapeutic applications recently explored the targeting of NK cells with memory functions, acquired during a previous activation (52, 53).

## 4 Memory-type NK cells

Under certain conditions, NK cells display adaptive and memory-like features against different pathogens, such as viruses, including HCMV (54), HIV (55), chikungunya (56), and influenza (57). These findings primed the interest of researchers to explore the NK cell memory potential for cancer treatment of hematologic malignancies and solid tumors. The ability of the immune system to remember and rapidly respond against prior-faced pathogens is defined as immunological memory, which is mainly considered a characteristic of adaptive immune cells. Recent studies indicate that innate immune cells can also exert memory function against a previously encountered pathogen (52, 53). Memory-like (ML) NK cells can ignore some inhibitory receptors, such as KIR, and exhibit higher expression of the activating receptors, such as NKG2D, NKp46, and DNAM-1, suggesting their potential effectiveness in recognizing additional tumor types (26). ML NK cells show various features, such as longer persistence, higher proliferation, and extended effector function (58), that provide superior host protection, higher expansion, and higher IFN-γ production than conventional NK cells (59). In humans, the increased output of IFN-γ by ML NKs was correlated to the expression of NKp46, CD94, NKG2A, and CD69 receptors, as well as the lack of CD57 and KIR receptors (56). Additionally, it was found that the inhibitory receptor NKG2A is a dominant checkpoint for the ML NK-cell phenotype, and that CD8α^+^ donor NK cells correlate to treatment failure against AML acute myeloid leukemia (AML) (60). We summarized the specific features of ML NK cells in comparison to classical NK features in **Table 3**.

**Table 3:** Types of memory-like NK cells.

NK cell type	Memory-Like	Phenotype	Transcription factors	Specific features
Traditional	No	CD3^−^CD56^+^ lymphocytes with different expressions of CD16 and CD56	• GATA2, EOMES, and T-bet commitment to transitional NK cells promote the transition from immature and mature NK cell stages (61).	• Natural cytotoxic activity. • Mediation of anti-viral and anti-tumor responses.
Hapten-induced	Yes	Expression of CXCR6 chemokine receptor (62).	• Aryl hydrocarbon receptor (AhR) is required for NK hapten-memory function (63).	• Long-lived immune memory cells. • Increased cytotoxicity.
Virus-induced	Yes	NKG2C^+^ NK cell subset found after viral exposure (64, 65).	• IRFB8 and BTB-ZF (Zbtb32) transcription factors and BNIP3- and BNIP3L genes are essential for adaptive NK cell antiviral immunity (25, 66, 67).	• Increased cytotoxicity. • Long-lived memory NK cell population.
Cytokine-induced	Yes	CD117 (c-Kit), CD127 (IL-7Rα), and CD122 (IL-2Rβ) cytokine receptors outline the stages of NK cells (61).	• STAT4 signaling is required to express several genes when *in vitro* cytokine-stimulated NK cells are generated. • STAT4 binding directly influences the expression and upregulation of IFN, STAT1, Zbtb32, Tbx21, Runx1, and Runx3 (68, 69).	• Increased cell sensitivity. • Reduced risks of irradiation and contamination during large-scale NK cell expansion.

The use of ML NK cells has been considered reliable and sufficient to stimulate remissions in patients with hematological malignancies such as AML and multiple myeloma (MM) patients (60, 70). Activated NK cells effectively targeted and killed compromised cells in pediatric and adult patients with acute lymphoblastic leukemia (ALL). Boieri et al. demonstrated that Roser leukemia was highly resistant to autologous NK cells. That resistance could be overcome using autologous NK cells pre-activated with IL-12, IL-15, and IL-18 cytokines *in vitro* and *in vivo* (71). Romee et al. demonstrated that ML NK cells induced with IL-12, IL-15, and IL-18 cytokines had highly functional responses, enhanced INF-γ production, and higher cytotoxicity when stimulated *in vitro* with primary human AML blasts and against several myeloid leukemia cell lines. Also, good clinical responses were observed in five of nine patients, including four complete remissions (72). The *in vitro* activation of ML NK cells was also shown to have a greater and longer capacity to contain the *in vivo* growth of multiple myeloma cell lines compared to IL-2 activated NK cells (73).

ML NK cells also exhibited promising *in vitro* results for treating solid tumors, encouraging their use in the early phases of clinical studies (see **Table 1**). For example, human ML NK cells were injected in NSG (NOD scid gamma) mouse models, demonstrating higher control of melanoma xenografts compared to conventional NK cells maintained with IL-15 (26). Uppendahl et al. studied the interaction of ML NK cells from healthy donors and melanoma patients against melanoma targets and established the superior cytokine production (IFN-γ) and cytotoxicity of ML NKs. They correlated that these ML NK-cell responses against autologous targets were related to the activatory receptors NKG2D and NKp46. ML NK cells also showed enhanced *in vitro* production of TNF-α and IFN-γ against different ovarian cancer cell lines, including SKOV-3, MA148, OVCAR5, and A1847, but also controlled the MA148 tumor growth of an *in vivo* xenogeneic mouse model (74). Moreover, ML NK cells isolated from hepatocellular carcinoma (HCC) patients and healthy donors were identified as potential effectors against HCC by trafficking to the liver to exert a cytolytic activity responsible for lower levels of spontaneous HCC formation (75). Tanzi et al. studied ML NK cells against solid tumors and demonstrated that these cells were able to lyse autologous tumors, including lung, liver, and peritoneum tumors. Of note, in this study, the antitumor activity of IL-12, IL-15, and IL-18 cytokine-activated NK cells was superior to that obtained with IL-2 stimulated NK cells (76). Another study demonstrated that ML NK cells cultured in the presence of IL-2 and IL-21 showed enhanced expansion and cytotoxicity against K562 cells. This enhanced bioactivity was correlated to the upregulation of the markers CD69 and CD25, along with phosphorylation of signal transducer and activator of transcription (STAT) 1 and 3 (68).

In summary, the use of memory-like NK cells has shown to be advantageous and a promising alternative to improve the current results obtained with NK cell immunotherapy. However, to translate these advantages into a clinical setting, it is imperative that efficient methods can be deployed for the large, stable production of these cells.

## 5 Targeting the immunosuppressive tumor microenvironment

**Figure 2:**
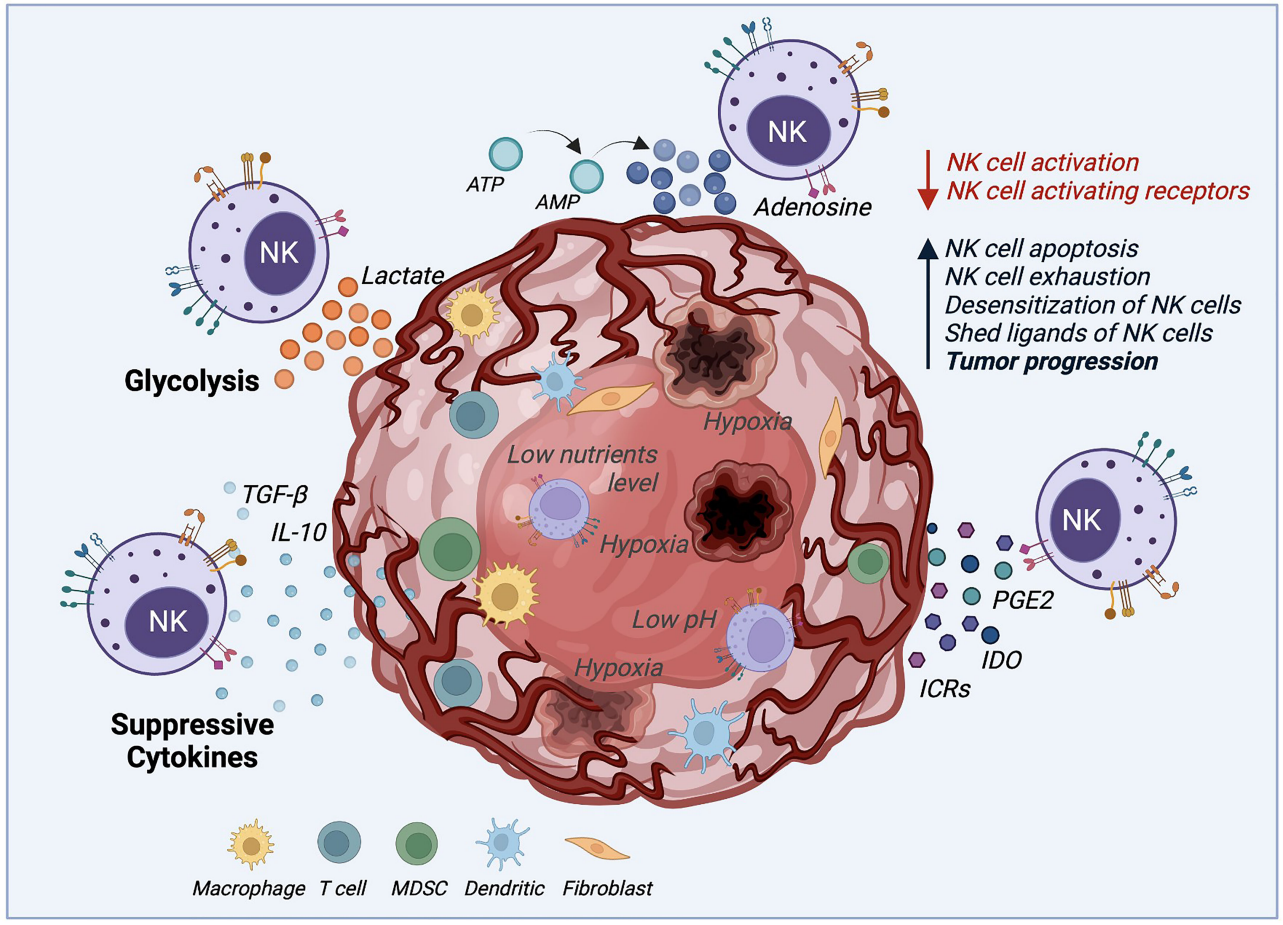
*Tumor microenvironment (TME) mechanisms inhibiting NK cell cytotoxicity.* The TME deploys several mechanisms to survive and increase the tumor progression rate by desensitizing NK cells and decreasing NK cell activation. These mechanisms negatively impact NK cell cytotoxicity by boosting NK cell apoptosis, exhaustion, and proliferation. Suppressive cytokines such as TGF-β and IL-10, immune-checkpoint receptors (ICRs), indoleamine 2,3-dioxygenase (IDO), prostaglandin E2 (PGE2) are secreted by the TME to desensitize NK cells. Also, the production of lactate and adenosine, along with hypoxia, and low levels of nutrients and pH, inhibits the NK cell function against solid tumors.

One of the significant challenges in using NK cell-mediated immunotherapy is the cells’ low activity when deployed against solid tumors, due to the several immunosuppressive mechanisms that the tumor microenvironment (TME) uses, as illustrated by **Figure 2**. The TME establishes immune evasion mechanisms that include acidification (low pH), hypoxia, decreased expression of tumor-associated antigens, and low nutrients. Also, the TME upregulates the expression of inhibitory ligands and downregulates transcriptional ligands complementary to NK cell-activating receptors (4, 77, 78).

For example, the activating natural killer group 2D (NKG2D) receptor is highly involved in NK cell-mediated tumor surveillance. Specific tumor cells developed a mechanism to evade NKG2D recognition by shedding and saturating the NKG2D receptor with NKG2D soluble ligand (79). This action, in addition to allowing cancer cells to hide from the effect of NK cells, also leads to the desensitization of NKG2D-mediated NK cell activation since high levels of NKG2D ligand shedding cause the downregulation of NKG2D signaling (80). Another immunosuppressive mechanism example is the increase of associated factors, including tumor-secreted factors (TSF) and tumor-secreted exosomes (TSE) (2). Some examples of TSE or tumor cytokine expression are the transforming growth factor (TGF)-β, tumor necrosis factor (TNF), vascular endothelial growth factor (VEGF), and IL-10 that induce the upregulation of key molecules for tumor cell proliferation and suppress the adaptive antitumor immune response (2, 4, 81). It was demonstrated that TGF-β leads to NK cell differentiation and reduces the expression of NK cell activating receptors NKG2D, NKp30, and DNAM-1, which is related to their lack of efficient activation (81, 82). Another factor that represents a challenge for NK cells, when facing cancer cells, is their ability to maintain normal MHC-I expression. This results in the inhibition of NK cell activation by binding to NKG2A and KIR (Killer cell Immunoglobulin-like Receptors). These receptors act as immune checkpoints, preventing NK-mediated cancer cell destruction (83).

Moreover, tumor metabolic activity, mitophagy, and oxygen availability also affect the NK cell responses (24). Tumor glycolysis can drive lactic acid accumulation in the TME. NK cells *ex vivo* culture with 15 mM lactic acid results in a totally blocked IFN-γ production, inhibiting NK cell proliferation and tumor surveillance in melanomas (84). Similarly, the mitochondrial fragmentation caused by hypoxia may be an essential factor to consider for NK cell therapy success (24). Zheng et al. found that mitochondrial fragmentation induced a loss of NK cell cytotoxicity, helping tumor evasion from NK cell-mediated surveillance (85). Hypoxia, along with nutrient shortage, acidic environment, abnormal vasculature, and high pressure, also plays an essential role in TME. Hypoxia not only stimulates the TSE release and attracts bone marrow-derived cells (BMDCs), but it may also increase neoplastic growth, slow down the tumor cell death rate, and promote cancer cells genomic variability leading to increased tumorigenesis (2). Similar to the TGF-β mechanism, hypoxia affects NK cell cytotoxicity by decreasing the expression of activating receptors NKp46 and CD16. Hypoxia can also induce overexpression of the adenosine nucleotidase CD73, upregulate checkpoint molecules such as PD-L1, and down-regulate the activating ligand MICA on tumor cells (51).

All of these survival TME mechanisms are challenges that autologous and allogeneic NK cells must overcome to be successful as an immunotherapy option. Lately, one alternative explored for therapeutic purposes is to target and boost NK cells that were previously activated to awaken their memory functions (52, 53).

## 6 Memory-like NK cell production

**Figure 3:**
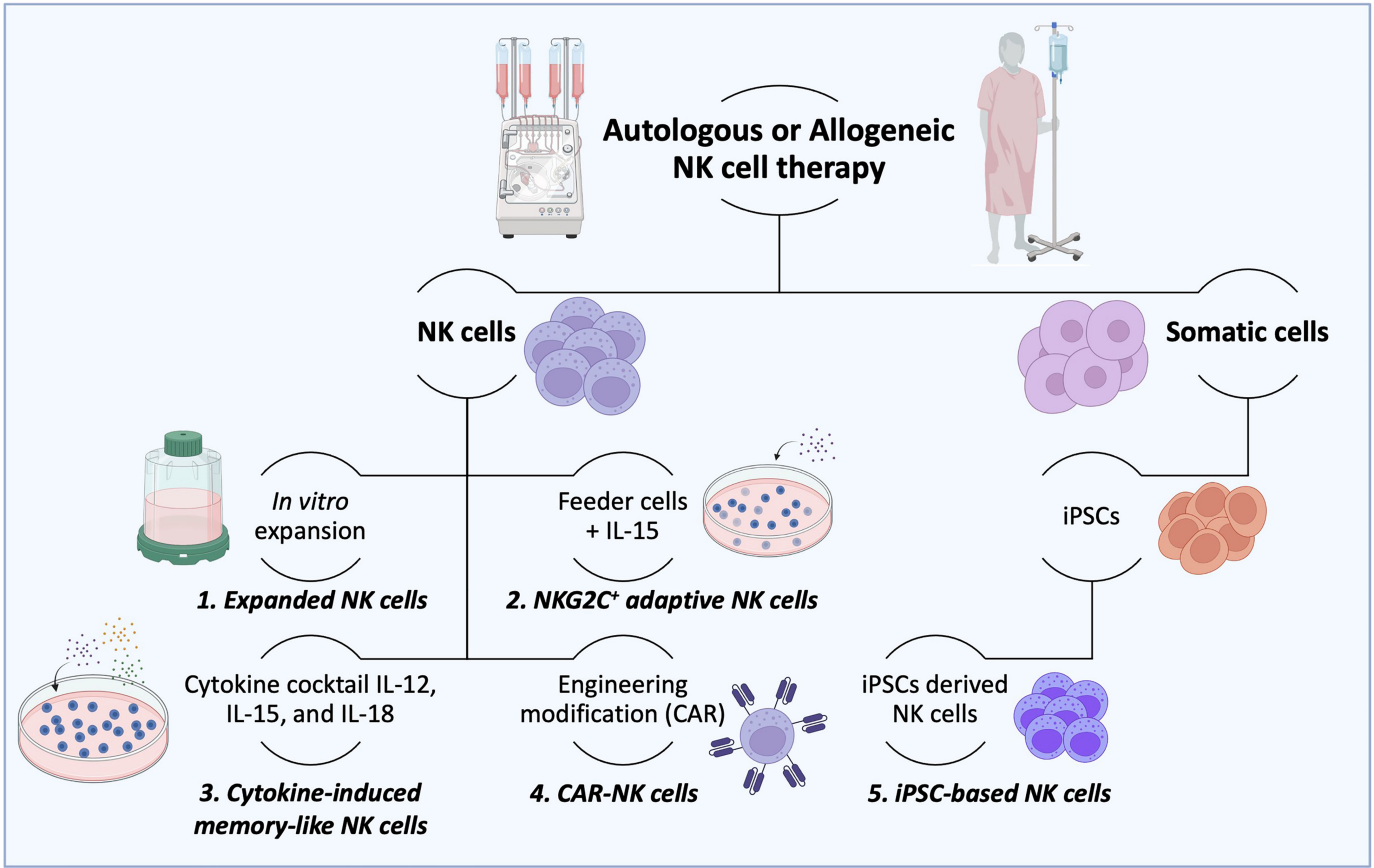
*Production of therapeutic NK cells.* For autologous or allogeneic clinical purposes, a considerable number of therapeutic NK cells are needed to destroy cancer cells effectively. To this end, several ways to obtain the desired number of therapeutic NK cells have been established. (1) After isolation, NK cells could be mass expanded *in vitro* in culture flasks, reactors, and bags until the needed cell number is achieved. (2) Irradiated feeder cells (to suppress their expansion) in the presence of IL-15 are employed to stimulate the NK cell activation and expansion. (3) The combination of cytokines such as IL-12, IL-15, and IL-18 is used to expand NK cells and generate memory functions (memory-like NK cells). (4) NK cells can also be potentiated by engineering modifications with CAR constructs to overcome their current limitations. (5) More recently, induced pluripotent stem cells (iPSCs) have been considered an advantageous source of NK and CAR-NK cells over peripheral and cord blood due to their usage in both autologous and allogeneic settings. (6) The IL-2-dependent immortalized NK-92 cell line is another type of NK cell source that possesses features of immune cell types and has been used in clinical trials.

The consensus is that innate memory forms in three stages (1). The cell expansion phase upon an activation event (encounter of the antigen/activator by naïve cells) (2); the contraction phase, when cells that return to baseline function undergo apoptosis; and survival cells enter (3) the memory phase, which produces enhanced response of remembering a prior-faced stimulation. However, NK cell memory differs from a classic adaptive memory, varying accordingly to the employed activation stimulus with unique functional and molecular characteristics (25, 86). Current strategies to produce a large number of NK cells are in development, with the hope of allowing the potential treatment of many patients from a single source, as observed in **Figure 3** (24). Several studies demonstrated that the generation of ML NK and memory-like responses could be achieved under specific circumstances such as hapten-specific exposure, viral infection, feeder cell exposure, and cytokine activation (25, 53).

ML NK cells obtained by antigen-specific exposure, were first described by O’Leary et al. when studying hapten-induced contact hypersensitivity (CHS) response in mice lacking T cells and B cells (87). Hapten-induced CHS is a typical example of adaptive immunity, where the epithelial surface is exposed to molecules that chemically modify proteins. Those “haptenated molecules” becomes foreign antigens triggering the formation of hapten-specific and long-lived immune memory cells. In this study, it was found that the Ly49C-I+ NK subpopulation localized in the donor’s liver exhibited responses specifically to DNFB (2,4-dinitro-1- fluorobenzene) and OXA (oxazolone) haptens that persisted for at least 4 weeks (87). After that, it was demonstrated that the CXCR6 (C-X-C chemokine receptor type 6) chemokine receptor plays a critical role in NK memory response to haptens and viruses. CXCR6 was present on hepatic NK cells and was responsible for their persistence and homeostasis, but not for antigen recognition. Although the CXCR6 receptor expression is not limited to the liver, only hepatic NK cells mediated the memory response (62). Another study of classical CHS protocols with immunocompromised mice that lacked T and B cells, but not NK cells, demonstrated that liver NK cells induced specific memory responses to haptens. This behavior was confirmed upon transfer in CD3-deficient mice, confirming the existence of “memory-like” NK cells (88). Consequently, it was established that DNFB-induced liver NK cells were potent effector cells that appeared within 1 hour after immunization. Authors suggested that hapten-specific effector NK cells are pre-existing in naive hosts, ready to exert inflammatory responses immediately after antigen contact (89). Antigen-specific NK cell memory was also found in primate species (rhesus macaques) after SIVmac251- and SHIV162P3 infections and AD26 vaccination, suggesting a potential use in vaccines against HIV-1 and pathogens (55). Likewise, it was found that specific subpopulations infected with human cytomegalovirus (HCMV) possess an NKG2C^+^ NK cell subset that exhibits clonal-like expansion and partially mimics an anti-viral adaptive response (64). Another study showed that NK cells exposed to therapeutic antibodies such as AFM13 (CD30/CD16A) produced more IFN-γ and showed increased cytotoxicity upon restimulation with lymphoma tumor cells, indicating memory-like functionality (65).

The use of feeder cells such as PBMCs, Jurkat T cells, K562 leukemia cells, and Wilms tumor HFWT have also been explored to produce ML NK cells. This method shows promising survival and growth results even for large-scale NK cell expansion. Feeder cells can promote cell growth by triggering the extracellular secretions of growth factors, detoxification of the culture media, synthesis of extracellular matrix proteins, or improved cell attachment (90). For example, NK cells expanded in a co-culture setting with irradiated (40 Gy) 721.221-AEH cells showed a high killing capability against acute lymphoblastic leukemia cells, that could be easily scaled up into a GMP-compliant process. Interestingly, even when the cytotoxicity response was optimal, less than 30% of the cell population showed CD107a marker upon degranulation, which was explained by the different killing mechanisms that natural killer cells could perform in response to the target (91). Jurkat T cells sub-line KL-1 induced a 100-fold NK cell expansion with almost 90% purity of NK cells from PBMCs, with potent antitumor capability. The *in vitro* and *in vivo* tumor-lytic capacity was associated with the expression of natural cytotoxicity receptors (NKp30, NKp44), activation receptors (NKG2D, DNAM-1), and adhesion molecules (CD11a, ICAM-1) (92). Authors also suggested that the positive results using feeder cells could be enhanced using genetically modified cells. Indeed K562-mb15-4-1BBL cells (K562 cells modified to express membrane-bound IL-15 and 4-1BBL) are already used in several clinical trials of allogeneic NK cells (93). Membrane-bound cytokines IL-2, IL-15, or IL-21 in K562 feeder cells, lacking HLA, but expressing costimulatory molecules, can be used for effective NK cell *ex vivo* expansion. Concurrently, Min et al. combined the use of irradiated autologous PBMCs and the monoclonal antibody OKT3 in the presence of IL-2 to large-scale NK cell expansion for 3 weeks and evaluated the effect of a freeze/thaw cycle. NK cells reached approximately 15,000-fold expansion, and while their viability was slightly reduced after thawing, their cytotoxicity and cytokine secretion against hepatocellular carcinoma cells were not affected (94). Rezaeifard et al., as part of an autologous phase I clinical trial, executed a pre-clinical NK cell enrichment employing anti-CD56 and anti-CD3 in PBMCs with the addition of IL-2. After two weeks, these expanded NK cells (CD3-CD16+/-CD56+) presented highly lytic properties against K562 cells, reaching a 510-fold average with a strong expression of NKG2D and CD16 (95).

Another method to support the activation, expansion, and survival of NK cells is the addition of cytokines to the culture medium, traditionally using IL-2 and, most recently, IL-15 (72, 96). The rationale behind using cytokines to stimulate NK cells is to increase cell sensitivity by targeting different costimulatory molecules and activating receptors (97). Various combinations and permutations of the cytokines IL-12, IL-15, IL-18, and IL-21 were shown to produce ML NK cells for pre-clinical and clinical studies. Terren et al. studied different combinations of IL-12, IL-15, and IL-18 cytokines to generate human cytokine-induced memory-like (CIML) NK cells. They found that IL-15 significantly contributes to NK cell cytotoxicity, but combining the three cytokines induced a synergistic effect that granted the cells the best polyfunctional profile. That profile includes proper degranulation and production of multiple cytokines and chemokines such as INF-γ, TNF-α, and CCL3 (C-C motif chemokine ligand 3) (98). Romee et al. developed the first-in-human phase I clinical trial of CIML NK cells with low dose IL-2, demonstrating high functional responses enhancing INF-γ production and cytotoxicity. This study showed promising results, with good clinical response in five of nine treated myeloid leukemia patients (72). Shapiro et al. performed a phase I clinical trial infusing allogeneic CIML NK cells in acute AML patients after lymphodepletion and subcutaneous IL-2 dosage. NK cells stimulated for 12-16 hours with the cytokine cocktail (IL-12, IL-15, and IL-18) and infused at a range of 5 to 10 million cells/kg demonstrated a rapid *in vivo* expansion and long-term persistence after infusion (59). Other research focused on the large-scale production of ML NK cells through cytokine stimulation. Liu et al. established a strategy to develop a high-efficient large-scale generation of NK cells from peripheral blood. In that study, NK cells stimulated with IL-2, IL-15, and IL-18 showed prolonged persistence, high cytotoxicity against K562 cells, and high levels of activating molecules such as CD16 and NKG2D (99). While feeder cells have previously been employed for large-scale expansion (100), the use of cytokine-induced stimulation could avoid the problems that come along with the use of feeder cells. Some examples of those problems include irradiation for inactivation, product contamination by feeder cells, and overall safety risk that can limit the large-scale production of NK cells for clinical purposes (99).

## 7 Enhancing NK cells potency against cancer

Current NK cells-mediated cancer immunotherapy produced encouraging results against hematological malignancies and solid tumors (1, 4). Still, these results can likely be potentiated in combination with other cell lines (101), nanoparticles (102–104), as well as chemotherapy and radiotherapy (105).

The combination of NK cell therapy with cytotoxic T cells was demonstrated to kill more cells than the sum of individual treatments. Notably, these 2 cell types are the most widely used lymphocytes in cancer immunotherapy, which aims to target cancer cell populations through opposites mechanisms, suggesting the existence of cytotoxic synergies (37, 101). Additionally, IFN-γ production of NK cells in the TME upregulates MHC-I expression by tumor cells, increasing activatory targets for cytotoxic T cells (106, 107). Therefore, combining NK cells with T cells is a potential mechanism to achieve tumor regression. For example, Zhang et al. studied this combined human lymphocyte treatment against primary tumor tissue of lung cancer *in vitro* and *in vivo*. They found that allogeneic NK cells were more cytolytic against cancer cells than autologous NK cells; activated T cells primarily impacted MHC class I-positive cancer cells, while NK cells focused on MHC class I-negative cancer cells (101). This result could be correlated to the fact that CD8^+^ T cell response and expansion increased with the presence of NK cells in the B16F10 melanoma (2). Friedmann et al. used melanoma cells as a cancer model to study the combinatorial cytotoxicity of NK cells and cytotoxic T lymphocytes (CTL). They found that after 24 hours of co-culture using high effector-to-target (E: T) ratios, melanoma cells were fully lysed, while at lower E: T ratios (simulating human cancer conditions), a significant number of melanoma cells survived and became more resistant to further NK cell exposure. Interestingly, this detrimental effect could be reversed if the initial NK exposure is followed by CTL exposure, which the authors suggest could be due to the release of exogenous antigens (108). The engineering modification of NK cells in combination with CAR-T cells has also been explored to enhance the potency of cell therapy treatments, showing that NK cells support the anticancer CAR-T cell response (109). In combination with CD19 CAR-T cells, cord blood-derived NK cells induced an enhanced antitumor efficacy of CAR-T cells against multiple myeloma, by facilitating their earlier activation and improving their migration to the tumor cells (109). It was also observed that in malignant mesothelioma patients, the physiological levels of CD56^Bright^ and CD56^Dim^ NK cell subset populations were abnormal. However, the addition of an anti-cytotoxic T lymphocyte-associated antigen 4 (CTLA4) checkpoint restored those NK-subset levels, activity, and killing capability (110).

NK cells have also been associated with other immune cells than T cell lymphocytes to enhance the cytotoxic response against cancer. In human melanoma, stimulatory dendritic cell abundance was found to be related to the intra-tumoral expression of the gene encoding the cytokine FLT3LG, which is principally produced by NK cells. Also, Böttcher et al. found that the interplay between NK cells and conventional type 1 dendritic cells (cDC1) has numerous therapeutic implications within the TME (111). For instance, intra-tumoral transcripts CCL5, XCL1, and XCL2 closely correlate with NK cells and cDC1 gene signatures that were also associated with patient survival (111). This cell interaction suggested an excellent tool for the success of cell immunotherapy since NK cells are required to promote the optimal cytotoxicity response of T cells through the recruitment of dendritic cells in the tumor site (21, 107, 111, 112).

Nanoparticles have also been explored to enhance NK cell therapy in multiple research groups (103). Magnetic (113), chitosan (114), lipid nanoparticles (115), liposomal polymeric gel (116), and PLGA microspheres (117) are examples of nanoparticle substrates employed in previous studies to augment NK cell immunotherapy against several types of cancers. Namely, Adjei et al. used manganese dioxide nanoparticles as a vehicle to deliver siRNA (small interfering RNA) for the NK cell TGF‐β receptor 2, which resulted in the restoration of NK cell activity against lung cancer cells (102). Most recently, Biber et al. developed a non-viral lipid nanoparticle to encapsulate and deliver small interfering RNAs (siRNAs) to overcome current obstacles in NK cell-based immunotherapies. They showed that their nanoparticles targeted NK cells *in vivo*, silencing their intrinsic inhibitory checkpoints and triggering NK cell activity to kill tumors (118).

Currently, the combination of chemotherapy with NK cell therapy is tested in multiple cancer clinical trials (see **Table 1**). This combination can be a synergistic strategy to improve outcomes in order to facilitate effector cell trafficking, cell infiltration, NK cell-mediated cytotoxicity, lysis, and cancer antigen release (105, 119). Several chemo-drugs also promote the expression of NK cell activating receptors and their ligands expression on tumor cells, and enhance the secretion of selected chemokines, making cancer cells more vulnerable to NK cell cytotoxicity (37, 105). A vast list of available drugs can be considered to potentiate the NK cell therapy results. Antitumor antibiotics, antimetabolic and alkylating agents, plant alkaloids, proteasome, histone deacetylase, and tyrosine kinase inhibitors are types of drugs commonly used in chemotherapy (105). That potentiation has been demonstrated with different types of cancer. For example, Bae et al. demonstrated the safety of a high-dose autologous NK cell therapy combined with chemotherapy in a phase I clinical trial. They employed 4 cycles of hepatic arterial infusion chemotherapy (HAIC) of 5- fluorouracil (750 mg/m2) and cisplatin (25 mg/m^2^) (48). Additionally, it was confirmed that combining NK cells with alkylating agents sensitizes melanoma cells, allowing the control of tumor growth. Using dacarbazine in melanoma patients showed an increased expression of the NKp46 receptor (120), and a higher NK cell activation and cytolytic activity (121). Another study revealed that using lymphodepletion chemotherapy before injecting NK cells in patients with AML combined with a low dose of IL-2, increased IL-15 production and promoted NK cell persistence and expansion (42). The chemotherapy regimen included cyclophosphamide and methylprednisolone, and fludarabine (42). Other types of cancer, including ovarian cancer (122) and advanced colon carcinoma (123), have also been treated with NK cells and chemotherapy combinations. Moreover, NK cells combined with chemoradiotherapy induced long-term tumor control against metastatic nasopharyngeal carcinoma (124). An intriguing avenue to enhance the cytotoxicity of NK cells in the TME is to use artificial engagers that both support NK function and lower the TME capacity for immune inhibition while lowering the potentials for side-effects. For example, a fusion complex combining an IL-15 agonist coupled with a TGF-β molecular sink showed increased infiltration of both NK and CD8+ cytotoxic cells and lower tumor burden in a syngeneic mouse B16F10 melanoma model (125).

Other combinatorial studies have also demonstrated additional avenues for potentiating the anti-cancer effect of NK-based therapies. For instance, it was reported that the combined treatment of NK cells with oncolytic viruses (OV, therapeutically beneficial viruses that have the ability to replicate and kill cancer cells) enhanced the cytotoxicity of OV and induced NK cell infiltration (107, 126–128). Another study found that using a checkpoint antibody blocking TIGIT, an inhibitory cell surface receptor, reversed the NK cell exhaustion and enhanced NK cell cytotoxicity, inhibiting *in vivo* colon, breast, and melanoma tumor growth (129).

In addition to the potentiated NK cell therapy combinations already mentioned, engineered modifications of NK cells is a fast-growing field. Further details are mentioned in the next section.

## 8 Engineered modification of NK cells

The genetic modification of NK cells to express CAR (Chimeric Antigen Receptor) constructs has recently gained significant attention, due to the success met using this technology with T cells. CAR were first described in 1987 (31) and are bioengineered cell receptors coded by “chimeric genes composed of immunoglobulin (Ig)-derived variable (V) regions and T-cell receptor (TCR)-derived constant (C) regions”, allowing for the specific of cancer cells while activating the engaged immune cell. Since NK possess several activation receptors, this concept was ideal to be used for the design of CAR-NK cells (130). These modifications intend to overcome the limitations of non-modified NK cells when facing tumor cells with genetic or epigenetic variations that bypass the NK immunological surveillance (51, 58). CAR-NK cells can be produced from multiple sources (such as peripheral blood, cord blood, iPSCs, and NK-92 cell line), adopting the basic methods borrowed from CAR-T cell generation (131). The CAR molecule, a synthetic hybrid antigen receptor (132), can redirect the NK cell-killing capability to a specific antigen target. The main characteristics of CAR-NK cells versus traditional NK cells have been summarized in **Table 4**.

**Table 4:** Comparison of NK and CAR-NK cell products.

Feature	NK cells	CAR-NK cells
Specificity	Do not have preferential targeting (targets any compromised cells).	Specific to target antigen expresses on the cancer cell surface. But many healthy cells also express cancer-associated antigens.
Killing mechanism	NK cell receptors mediate the cell-killing capability.	NK cell receptors mediate the cell-killing capability.
Gene editing/CAR	N/A	After modification, cells maintain their natural ability to detect stress-evoked ligands. Low genetic transfection levels <40% were obtained compared with CAR-T cells.
Dosage*	A single dose of 1x10^7^ to 1x10^8^ cells. NCT03383978. One to three cycles of 3-5x10^7^ cells/kg. NCT00855452. Daily scalable dosage 2.5×10^8^, 5×10^8^, 10×10^8^ NK cells. KCT0003973	Weekly scalable dosage (3x10^6^, 1x10^7^, 3x10^7^, 1x10^8^ cells). NCT02439788. Weekly infusion of 2x10^9^ cells. NCT04847466.
Manufacturing	Therapeutic NK cell expansion could take 2 to 5 weeks (up to the expansion method).	Additional time and cost to engineering expanded NK cells. After engineering, a lower survival cell rate is observed, and it takes additional recovery time before using cells.

*Dosage based on the available information on glioblastoma, neuroblastoma, and gastric or head/neck treatments. Source: https://clinicaltrials.gov.

To date, NK cells have been engineered and modified with CARs in order to overcome challenges such as the limited persistence after infusion without cytokine support, slow trafficking to tumor beds, and overcoming the immunosuppressive TME (131). Similar to CAR-T cells, CAR-NK cell functionality was initially tested against hematological malignancies. In a pre-clinical study, the combination of CIML NK cells expressing an anti-CD19 specific CAR was tested to enhance the response rate in NK-resistant cancers. Results demonstrated an increased functional response (degranulation, IFN-γ production, and specific killing) against B cell lymphomas by CD19-CIML NK cells compared with CD19-NK, CD33-NK, and ML NK cells (133). These results underline the great potential that CAR-NK cell therapy could offer compared with non-modified NK cells. In addition to CD19, there are almost 50 other surface target antigens, including CD5 (134), EGFR or EGFRvIII (135–138), HER-2 (139–142), NKG2D (143–146), and GD2 (147, 148) that have been considered to enhance CAR-NK cell responses (149).

Additionally, it is known that NK cells are characterized by a short-lived persistence in the body after infusion in the absence of cytokine support, approximately 14 days, which limits the efficacy of NK cell immunotherapy. That limitation was intended to be overcome by the genetic modification of NK cells with transgenes encoding cytokines IL-2 or IL-15 either for secretion or membrane expression. This strategy yielded the suppression of inhibitory signals of the TME as well as enhancing the NK cell killing capability in several studies (131, 150, 151). Also, to modulate the CAR-NK cell functionality, the expression of cytotoxic ligands and chemokine receptors such as CXCR4, EGFRvIII, and the combined expression of FRα and DR4/5 were included in the engineering modification design (137, 152, 153). The NK cell metabolism, cell homing, and tumor infiltration were also enhanced with engineering modification (154). For example, to improve the metabolism within the TME, maintaining the levels of cMyc signaling with kinase glycogen synthase kinase-3 (GSK3) inhibitors (155, 156), as well as the deletion of hypoxia-inducible factor 1a (HIF1a) (157) were a target of modifications. Cell homing is another critical factor considered in the NK cell therapy success, which was suggested to be targeted with different mechanisms (154). For example, lymphodepletion complemented with cytokine support (158), expression of the CXCR4 gene (152), pharmacologic combination (139, 159), and others were employed, having encouraging results in cell homing that also impacted the cell trafficking and tumor infiltration.

The first clinical trial of CAR-NK cell treatment focused on CD19 as a target (see **Table 5**), which showed a 73% positive response rate without evidence of GvHD, development of cytokine release syndrome, or neurotoxicity. Infused CD19-CAR-NK cells also demonstrated expansion and persistence for at least 12 months at low levels (160). After that trial, other phase I and II clinical studies were performed in USA and China. Some of these studies focused on hematological malignancies such as lymphomas (13 trials) and multiple myeloma (NCT05182073, NCT03940833). Others focused on solid tumors (131), including colorectal (NCT05213195, NCT05248048), glioblastoma and neuroblastoma (NCT03383978, NCT02439788), prostate (NCT03692663), ovarian (NCT03692637), metastatic gastric or head and neck cancer (NCT04847466), and pancreatic (NCT03941457) cancer. Some of these studies were withdrawn because of funding issues (NCT03579927) and methodology improvement (NCT02439788), while others have unknown statuses (NCT03940833, NCT03941457, NCT03692637, NCT03415100, NCT03940820).

**Table 5:** Current CAR-NK cells clinical trials.

Identifier	Cancer type	Target	Additional treatment	Recruitment Status	Phase	Goals and Outcome
NCT05020678	B cell cancers	CD19	NA	Recruiting	Phase I	Determine the incidence of treatment-emergent adverse events and the proportion of subjects experiencing dose-limiting toxicities of NKX019 therapy. No results have been posted.
NCT04623944	Hematological Malignancies or Dysplasias	NKG2D ligands	Cyclophosphamide, Fludarabine, Cytarabine (ara-C)	Recruiting	Phase I	Determine the incidence of treatment-emergent adverse events and the proportion of subjects experiencing dose-limiting toxicities of NKX101 therapy. No results have been posted.
NCT05215015	Acute Myeloid Leukemia	Anti-CD33/CLL1	NA	Recruiting	Early phase I	Determine the incidence of DLT within each dose, 28 days after administration. No results have been posted.
NCT03056339	B Lymphoid Malignancies	CD19-CD28-zeta-2A-iCasp9-IL15	Fludarabine, Cyclophosphamide, Mesna, AP1903	Active, not recruiting	Phase I and II	Determine the optimal NK Cell dose level, toxicity, and efficacy of cell product within 45, 14, and 30 days after infusion, respectively. No results have been posted.
NCT05379647	B-Cell Malignancies	CD19	QN-019a, Rituximab, Cyclophosphamide, Fludarabine, VP-16	Recruiting	Phase II	Determine the incidence of subjects with DLT within each dose level cohort and treatment-emergent adverse events (28 days). No results have been posted.
NCT05092451	Relapse/Refractory Hematological Malignances	CAR.70/IL15	Cyclophosphamide, Fludarabine phosphate	Not yet recruiting	Phase I and II	Determine the number of participants with treatment-related adverse events, complete or partial response, and who are alive and in remission. No results have been posted.
NCT05487651	B-Cell Malignancies	CD19	NA	Not yet recruiting	Phase I	Determine the incidence rate and the grade (severity) of DLTs based on adverse events (AEs). No results have been posted.
NCT04288726	Lymphomas	CD30	NA	Recruiting	Phase I	Determine the DLT rate. No results have been posted.
NCT04952584	Lymphomas	CD30	NA	Not yet recruiting	Phase I	Determine the DLT rate. No results have been posted.
NCT03579927	B-cell Lymphoma	CD19-CD28-zeta-2A-iCasp9-IL15	Carmustine, Cytarabine, Etoposide, Filgrastim, Melphalan, Rituximab,	Withdrawn (Lack of Funding)	Phase I and II	Determine the incidence of adverse events, and CR or PR. No results have been posted.
NCT05182073	Multiple Myeloma	BCMA	Cyclophosphamide, Fludarabine, Daratumumab	Recruiting	Phase I	Determine the incidence and nature of adverse events and DLTs, along with the RP2D. No results have been posted.
NCT03940833	Multiple Myeloma	BCMA	NA	Unknown	Phase I and II	Determine the occurrence of treatment related adverse events. No results have been posted.
NCT05213195	Refractory Metastatic Colorectal Cancer	NKG2D	NA	Recruiting	Phase I	Determine the DLT (safety) and MTD (tolerability evaluation). No results have been posted.
NCT05248048	Metastatic Colorectal Cancer	NKG2D	NA	Recruiting	Early phase I	Determine the DLT (safety) and MTD (tolerability evaluation). No results have been posted.
NCT04847466	Metastatic Gastric or Head and Neck Cancer	PD-L1	Pembrolizumab, PD-L1 t-haNK	Recruiting	Phase II	Determine the response rate with irradiated PD-L1 CAR-NK cells in combination with N-803 plus pembrolizumab in enrolled patients. No results have been posted.
NCT03383978	Glioblastoma	HER2	Ezabenlimab	Recruiting	Phase I	Determine the number of participants with treatment-related adverse events, MTD or MFD, and period of detectability of NK-92/5.28.z cells in blood and cerebrospinal fluid. No results have been posted.
NCT02439788	Neuroblastoma	GD2	Cyclophosphamide, Fludarabine	Withdrawn	Phase I	Determine the number of patients with DLT within 4 weeks. No results have been posted.
NCT03692663	Prostate Cancer	PSMA	Cyclophosphamide, Fludarabine	Recruiting	Early phase I	Determine the occurrence of treatment related adverse events. No results have been posted.
NCT03941457	Pancreatic Cancer	ROBO1	NA	Unknown	Phase I and II	Determine the occurrence of treatment related adverse events. No results have been posted.
NCT03692637	Epithelial Ovarian Cancer	Mesothelin	NA	Unknown	Early phase I	Determine the occurrence of treatment related adverse events. No results have been posted.
NCT03415100	Metastatic Solid Tumors	NKG2D	NA	Unknown	Phase I	Determine the number of treatment adverse events and anti-tumor response due to CAR-NK cell infusions. No results have been posted.
NCT05194709	Advanced Solid Tumors	5T4	NA	Recruiting	Early phase I	Evaluate the safety and tolerability of anti-5T4 CAR-NK cells. No results have been posted.
NCT03940820	Solid Tumors	ROBO1	NA	Unknown	Phase I and II	Determine the occurrence of treatment related adverse events. No results have been posted.

RP2D, highest dose with acceptable toxicity; MFD, maximum feasible dose; CR, complete response; PR, partial response. Source: https://clinicaltrials.gov.

Nevertheless, before translating the use of CAR-NK cells in clinical trials, some challenges must be addressed. Their limited persistence/expansion and their difficulty in being genetically modified must be addressed (152, 154, 161, 162). Some examples of successful genetic modification include the work of Tang et al., which reached over 90% transduction efficiency of CD33-CAR construct in the NK-92 cell line (163). But, NK cells are generally heterogeneous and have proven to be more challenging to expand and engineer *in vitro* by transduction, transfection, or nucleofection than T cells. During and after the engineering process, they show high sensitivity to apoptosis and low gene expression levels, which implies instability of the product and that the cost of obtaining therapeutic CAR-NK cells could be significantly higher than other alternatives (164).

The CAR transduction techniques employed for NK are primarily based on viral transduction and non-viral mediated transfection. The latter is the preferred method as it will induce fewer toxicities (149). The most successful current techniques yield a rapid, high, but transient expression (electroporation) or a sustained but low gene expression (viral vectors) (165). Despite the limitations, the most successful non-viral gene delivery technique in NK cells is the rapid transient expression by electroporation (166). Electroporation yields highly efficient T-cell transfection, but that result was not observed at a similar level with NK cells (154). For example, in terms of engineered modification of blood-derived NK cells, lipid-based and electroporation methods typically result in less than 5% transfection; retrovirus vector-based yield a 27-50% efficiency, and lentivirus-based transfection only yields a 20-40% efficiency (167). Nevertheless, even when viral transduction produces better results, they are associated with decreased cell viability, NK cell apoptosis, and reduced efficacy (154). To date, some *in vitro* and *in vivo* attempts at genome editing techniques aim to design suitable CAR-NK cell products (168). For example, the NK cells modification with a chimeric receptor consisting of activating receptor NKG2D (143), DNAX-activation protein 12 (DAP12) with CD3ζ (169), and NKG2D-ζ (170) were used to improve NK cell cytotoxic activity. But even when CAR-NK cells are designed to enhance further the results obtained with normal NK cells, the use of engineering NK cells does not always appear to produce optimal results. For example, Bachiller et al. studied the therapeutic effect of CAR-T cells with CAR-NK cells and NK cells combinations against non-Hodgkin’s lymphoma (NHL) and multiple myeloma (MM) using cord blood as an NK cell source. The effectiveness of CAR-NK cells was observed only when using higher doses of cells, with a rapid loss of activity over time compared with CAR-T cells, while the control NK cells produced a synergistic anticancer CAR-T cell response (109).

## 9 Challenges and future perspectives

The principal feature of NK cells is their natural ability to protect the host by recognizing, directing, and executing the rapid lysis of cancerous, stressed, and viral-infected cells without dependent priming (33–35). However, several challenges overshadow the effective NK cell-mediated cancer immunotherapy. Those challenges, including short-term persistence, sensitivity, immunosuppression of the TME, and clinical-grade *ex vivo* expansion, should be addressed for a successful cell therapy treatment (24). The potential for NK therapies, demonstrated in pre-clinical studies, has clearly supported sustained research but outside of the field of hematological cancers, very little to no progress have been reported in the clinic for any type of NK therapies against solid tumors with mostly due to a lack of results or early discontinuation of the trial. These discontinuations are in majority dues to either off-target toxicities or a lack of response. While it seems that NK-based immunotherapies are of very little benefit to the patient, it is worth looking at the potential for optimizations that might be responsible for some of these early lackluster results.

NK cells have a highly sensitive response to external stresses, such as cryopreservation. The wellness, stability, and proper therapeutic responses of NK cells throughout the entire manufacturing process are highly dependent on maintaining stable, stress-free physiological settings (171). NK cells are very susceptible to stress stimuli, particularly cryopreservation, which is reflected in their loss of viability and cytotoxic function after thawing (131, 167): the average rate of cryopreserved NK cell survival is less than 50%. Even when the cytotoxicity of viable cells can be recovered overnight in the presence of IL-2, recovering the pre-freezing cell numbers could take several days of *in vitro* expansion. Similarly, exposure to low temperatures, such as overnight refrigeration, affects cytokine-activated NK cells, as observed by a reduction of their cytotoxicity, even when their viability is not affected (167). In a recent study, cryopreserved NK cells demonstrated a 5.6-fold reduced killing activity against K562 tumor cells embedded in a 3D collagen matrix compared with fresh NK cells. This behavior may be explained by two mechanisms affected by cryopreservation: (1) loss of NK cell activation through cleavage of CD16, and (2) reduction of NK cell motility, preventing contact with target cells (172). Other factors affected by cryo-injury in NK cells are cell metabolic activity, cytokine activation, cell culture density, and persistence (131). Since this particular issue negatively impacts (through the use of cryopreservation, long-term storage, and shipping process) general immunotherapies before infusion, it is essential to intensify efforts and optimize procedures to reduce the NK cell dysfunction after thawing. Preserving the viability and cytotoxic function post-thaw is critical in a clinical setting to ensure a high consistency and quality of the products. Due to these issues observed after thawing during the *in vitro* culture, the direct infusion of freshly expanded NK cells “warm chain” may be advantageous. Another advantage to such “warm chain” would be the capability to add other immune fractions that could benefit from minimal handling and that can work in synergy with NK cells. γδT cells or CD8+ cytotoxic cells would be prime candidates for such combinations and could be obtained for the same leukapheresis product, for example. However, the realization of such personalized, combinatorial immune cell therapy would require the creation of a structure allowing the harvesting, isolation, expansion, re-invigoration and formulation of the immune cells at the point of care.

Regarding engineering modification, challenges such as the difficulty of transferring genetic material into NK cells in pre-clinical studies, selecting the most suitable and effective technique to accomplish the GMP grades should be a priority before scaling up the CAR-NK cell production. One of the primary issues with CAR-NK stem from either their lack of efficacy, or too much off-target cytotoxicity when applied in the clinic setting. These issues have been intensively scrutinized and researchers have been considering many variables to optimize therapies (149, 173, 174): (i) different cell sources such as immortalized cell lines (NK-92 or YT cell lines, that require irradiation before injection), peripheral NKs, stem-cell, umbilical or placenta-derived NKs, and ML-NKs, (ii) CAR design and use of the correct promoter, (iii) transduction methods such as retroviruses or electroporation. While these approaches have yielded encouraging pre-clinical results, it is still unclear as to how this can translate into the clinical setting. As underlined in **Table 5**, the lack of positive progress report or the discontinuation for current clinical trial are a testament of the difficulties to port these therapies to the clinic. Additionally, engineering of NK cell carries an additional production cost compared with non-engineered NK cells, and in some cases, the therapeutic response does not have the best cost-effectiveness ratio. Nevertheless, using NK cells over T cells as a vehicle to carry CAR expression has several advantages, such as the different NK cell sources and the donor–patient compatibility, which could reduce the average therapy manufacturing cost. Using iPSCs to obtain NK cells is a promising alternative to producing a significant number of therapeutic NK cells and then genetically modifying them. They are considered an advantageous source of NK and CAR-NK cells over peripheral and cord blood due to their versatility, homogeneity, high proliferation capacity, and usage in both autologous and allogeneic settings (4). This alternative is currently addressed in several research groups. For example, in 2019, Fate Therapeutics Company published clinical data about an iPSC-derived CAR-NK cell therapy against B Cell malignancies. In combination with rituximab, this therapy demonstrated prolonged *in vivo* cell survival and enhanced tumor-killing capacity compared with rituximab therapy alone (4, 175). Moreover, against solid tumors (e.g., ovarian cancer), iPSC-derived CAR-NK also showed enhanced anti-tumor activity, inhibition of tumor growth, and prolonged survival compared with iPSC-NK cells, T-CAR-iPSC-NK cells, or PB-NK cells (176). These results underline the great potential that iPSCs-derived CAR-NK cells could offer for cancer immunotherapy. One intriguing possibility would be to combine the high cytotoxic and survival of ML-NK cells with the increased specificity and targeting conferred by CAR modifications. However, while this combination might yield significant clinical effect, it is unclear how the downside of CAR modifications would ultimately negatively affect the outcome.

While significant technical progress has been made to enhance the properties of NK cells, most of the progress has sought to correct issues that stem from the allogeneic and bioengineered nature of current NK cell therapies: short persistence of the product and off-target cytotoxicity are instances described in this review. Additionally, we reviewed evidence here that at least some cell anergies could be linked to a decentralized process, introducing detrimental inefficiencies such as product lead time, shipping, and freezing/thawing. Typically, a cell therapy designed by a biopharmaceutical entity would have to be produced and shipped to a clinical site specialized in infusion, where the processes specific to the product needs to be executed. These processes are extremely specific, from shipping/receiving to handling and dosing, and in return require extreme expertise from the staff involved. Meanwhile, the possible complications linked to novel NK cell therapies will always require the availability of specialized post-care monitoring and care by a hospital system or a primary oncologist. However, both the medical and regulatory fields have been slow to recognize NK cells and their unique abilities (and cell therapy as a whole) as a new class of therapies, so it follows that only highly trained and specialized staff should be monitoring and managing patients for these early therapies and their specific needs. The fragmentation of the process, and lack of specialization of the involved structures (especially the hospital system), are clear hurdle to the obtention of stable and interpretable results. The slow progress of NK cell immunotherapy, and the *de-facto* focus on allogeneic use of the cells that can only exacerbate possible cytotoxicities, may be linked to the inefficiencies listed above. These problems could be addressed with a new clinical paradigm, where the patient’s cells could be directly harvested, expanded, and administered at the point of care, removing inefficiencies and lowering the cost of therapy (177), which remains one of the highest obstacles to the universal adoption of cellular immunotherapies. Patient’s safety and deviations would consequently be minimized and improve trials success and patient care. Even if this model could counteract to some of the downsides of CAR-NK production, it would still not answer the lackluster clinical observations from CAR-NK therapies in the solid tumor space. Autologous therapies and patients would be the main beneficiaries of this model, since the need for an “off-the-shelf” product would become obsolete as safe and longer-lasting therapies can be created on-site directly from the patient and become a prime solution for cancer immune cell therapies.

## Author contributions

RW contributed to the conception, design, revision and approval of this manuscript. GL-V contributed to the writing of the sections and the design of figures in the manuscript. MT-L, RD and JP contributed to the manuscript revisions and approved the submitted version. All authors contributed to the article and approved the submitted version.
